# Predictive Factors of Long-Term Survival after Neoadjuvant Radiotherapy and Chemotherapy in High-Risk Breast Cancer

**DOI:** 10.3390/cancers14164031

**Published:** 2022-08-20

**Authors:** Jan Haussmann, Wilfried Budach, Carolin Nestle-Krämling, Sylvia Wollandt, Balint Tamaskovics, Stefanie Corradini, Edwin Bölke, David Krug, Tanja Fehm, Eugen Ruckhäberle, Werner Audretsch, Danny Jazmati, Christiane Matuschek

**Affiliations:** 1 Department of Radiation Oncology, Heinrich Heine University, 40225 Dusseldorf, Germany; 2 Department of Gynecology and Obstetrics, Evangelisches Krankenhaus Dusseldorf, 40217 Dusseldorf, Germany; 3 Department of Senology, Sana-Kliniken Duesseldorf-Gerresheim, 40625 Dusseldorf, Germany; 4 Department of Radiation Oncology, University Hospital, Ludwig-Maximilians University (LMU), 80366 Munich, Germany; 5 Department of Radiation Oncology, University Hospital Schleswig-Holstein, 24105 Kiel, Germany; 6 Department of Gynecology and Obstetrics, Heinrich Heine University, 40225 Dusseldorf, Germany; 7 Department of Senology and Breast Surgery, Breast Center at Marien Hospital Cancer Center, 40479 Dusseldorf, Germany

**Keywords:** neoadjuvant radiotherapy, neoadjuvant chemotherapy, pCR, breast cancer, overall survival

## Abstract

**Simple Summary:**

This retrospective analysis reports on the treatment outcomes of women diagnosed with high-risk breast cancer treated with chemotherapy in combination with radiotherapy before the surgical removal of the tumor. It is well established that the lack of visible tumor cells in the pathological tumors analysis by the time of surgery (known as pathological complete response, pCR) is a factor that improves survival without the tumor reappearing in the body. However, it is unknown whether that is only true when giving systemic therapy or when pCR is achieved with the help of radiotherapy. We collected patient information and survival times to analyze the outcome in our patient group. We found that women with a pCR treated with chemotherapy in combination with radiotherapy can expect favorable long-term survival. This was true across different types of breast cancer and chemotherapy substances.

**Abstract:**

Background: Neoadjuvant radiotherapy (naRT) in addition to neoadjuvant chemotherapy (naCT) has been used for locally advanced, inoperable breast cancer or to allow breast conserving surgery (BCS). Retrospective analyses suggest that naRT + naCT might result in an improvement in pathological complete response (pCR rate and disease-free survival). pCR is a surrogate parameter for improved event-free and overall survival (OS) and allows for the adaption of the post-neoadjuvant therapy regimens. However, it is not clear whether pCR achieved with the addition of naRT has the same prognostic value. Patients and methods: We performed a retrospective re-analysis of 356 patients (cT1-cT4/cN0-N+) treated with naRT and naCT with a long-term follow-up. Patients underwent naRT on the breast and regional lymph nodes combined with a boost to the primary tumor. Chemotherapy with different agents was given either sequentially or concomitantly to naRT. We used the Cox proportional hazard regression model to estimate the effect of pCR in our cohort in different subgroups as well as chemotherapy protocols. Clinical response markers correlating with OS were also analyzed. Results: For patients with median follow-ups of 20 years, 10 years, 15 years, 20 years, and 25 years, OS rates were 69.7%, 60.6%, 53.1%, and 45.1%, respectively. pCR was achieved in 31.1% of patients and associated with a significant improvement in OS (HR = 0.58; CI-95%: 0.41–0.80; *p* = 0.001). The prognostic impact of pCR was evident across breast cancer subtypes and chemotherapy regimens. Multivariate analysis showed that age, clinical tumor and nodal stage, chemotherapy, and pCR were prognostic for OS. Conclusion: NaCT and naRT prior to surgical resection achieve good long-term survival in high-risk breast cancer. pCR after naRT maintains its prognostic value in breast cancer subtypes and across different subgroups. pCR driven by naRT and naCT independently influences long-term survival.

## 1. Introduction

Neoadjuvant or preoperative systemic therapy is the current standard treatment paradigm in higher-risk breast cancer [1,2,3,4,5]. Systemic cytotoxic targeted HER2-therapy and immune checkpoint inhibitors (ICI) are given to reduce macro- and microscopic disease downstage of the primary tumor to allow for less invasive surgery, reduce postoperative complications, and improve cosmetic outcomes. Adjuvant radiotherapy (RT) to the remaining breast tissue, chest wall, or draining regional lymph nodes is administered selectively according to the respective risk profile and response to the systemic treatment [6].

Neoadjuvant radiotherapy (naRT) was first introduced to downstage an unresectable advanced breast tumor to allow for an operation [1,7]. With the advent of breast-conserving surgery (BCS), naRT was also investigated with the aim to improve BCS rates [1].

Numerous trials have also tested the hypothesis that neoadjuvant systemic therapy (naST) allows for an earlier initiation of systemic treatment in higher-risk patients, which might improve survival due to the eradication of distant disease. A meta-analysis of randomized trials comparing neoadjuvant to adjuvant chemotherapy showed equivalent distant recurrence and breast cancer all-cause mortality rates [2]. However, naST enables the eradication of all invasive tumor cells at the time of resection (pathological complete response, pCR) which has been shown to correlate positively with survival [3,4,5,8].

Beyond the well-established indications, it has been hypothesized that administering radiotherapy in the neoadjuvant setting could further improve pCR and disease-free survival (DFS). A randomized trial and multiple retrospective analyses suggest a benefit for naRT over postoperative RT in terms of DFS and overall survival (OS) [9,10,11,12] but are not conclusive and not applicable to current treatments of breast cancer.

A cohort treated from 1990 to 2003 at the department of radiation oncology at the Duesseldorf University Clinic consisting of a mixed cohort of patients with high-risk or locally advanced tumors requiring neoadjuvant therapy, patients desiring a BCS, as well as women with an anticipated unfavorable cosmetic outcome due to tumor location or high tumor-to-breast ratio. We supplemented the previous cohort with 41 additional patients.

Previous publications by our group have reported possible increases in OS and DFS with neoadjuvant therapy [13]. Further, we showed that pCR was prognostic for OS and more likely to occur in smaller tumors and with increasing intervals to surgical resection [14].

The prognostic value of achieving a pCR with systemic treatment is well established. However, the question of whether a pCR achieved with the addition of a local therapy such as radiotherapy has the same prognostic value as a pCR achieved by systemic therapy alone is not known. We performed a re-analysis with a long-term follow-up and a reassessment of multiple factors to address this question.

## 2. Materials and Methods

We retrospectively revaluated the long-term outcomes of the cohort treated at the University Clinic of Duesseldorf, Germany and the Gerresheim Hospital in Duesseldorf, Germany from 1990 to 2003. The cohort has been described in more detail before [13,15,16,17]. The currently described cohort was supplemented by additional patients and a reassessment of different factors such as cytotoxic chemotherapy, reevaluation of pCR using a current definition, and updated survival status.

A total of 356 patients received naRT and chemotherapy before their definitive breast cancer surgery. Resection was performed as either a breast-conserving surgery with or without additional flab support or a mastectomy with or without reconstruction.

Clinical nodal assessments were obtained with use of clinical examinations and axillary sonography. Axillary lymph node dissection was routinely performed in all patients. Tangential radiation therapy of the breast was applied using photon or cobalt therapy. Regional nodal irradiation to the level III and IV axillary nodes and the internal mammary nodes (IMN) was applied in selected patients. Axillary levels III and IV were treated with a separate supraclavicular field and IMNs were covered by an extension of the tangential breast fields. The dose was mainly 50 Gy in 25 fractions to the breast with either a 10 Gy boost in 5 fractions to the tumor bed given with electrons, photons, or an interstitial HDR brachytherapy boost of 10 Gy in one treatment. Brachytherapy was combined with one course of hyperthermia immediately before interstitial treatment. Equivalent doses of 2 Gy (EQD2) were calculated using an alpha/beta ratio of 3.7 [18].

Neoadjuvant chemotherapy (naCT) was given either sequentially (mostly before RT) or concurrently to RT with multiple regimes. The systemic therapy regimen was decided by the interdisciplinary team evaluating the patient and based on standard protocols, individual risk factors, as well the patient’s response to ongoing therapy with clinical- and ultrasound-guided restaging. According to the pathological outcome, the interdisciplinary team also advise selected patients to undergo post-neoadjuvant systemic therapy. For the analysis, chemotherapy schedules were categorized according to the current known efficacy into “standard” regimes (AC/EC + taxane, AC/EC + CMF, and AC/EC + taxane + mitoxantrone) or “substandard” regimes (mitoxantrone only, AC/EC only, AC/EC + mitoxantrone, CMF ± mitoxantrone, and other rarely used regimes). Patients with hormone receptor-positive tumors received endocrine therapy with tamoxifen, ovarian suppression, aromatase inhibitor, or surgical ovarectomy. No HER2-targeted therapy was administered.

Biological breast cancer subtypes were defined according to hormone receptor status (estrogen or progesterone) and HER2 positivity or lack of positivity for both receptors (triple negative). Retrospectively, the hormone receptor status was assessed by immunohistochemistry with cutoff values greater than 10 fmol/mg of protein regarded as positives [19]. HER2-positive breast cancer was subcategorized accordingly into hormone receptor-positive (HR+/HER2+) or hormone receptor-negative (HR−/HER2+) subtypes. The hormone receptor-positive and HER2-negative subtypes were further categorized into luminal A-like and luminal B-like subtypes according to grading, estrogen and progesterone receptor status, as well the Ki-67-value. Grade I and grade II tumors with ER and PR expression above 20% and Ki-67 values below 14% were categorized as luminal A-like [19,20,21,22,23].

### 2.1. Endpoint Definition

We defined pCR as the state when there are no residual tumor cells in the lymph nodes or the breast/chest wall with residual component strictly in situ (ypT0/is ypN0) according to Chevallier’s classification [24]. “Any primary tumor downstaging” was defined as any reduction in tumor stage between the clinical assessment before treatment initiation and the pathological assessment at the time of surgery. The mean tumor size reduction was the maximum diameter measured in the ultrasound subtracted by the maximum diameter assessed in the pathological analysis. For the evaluation of the residual tumor volume, we idealized a spherical tumor volume from the measured diameter. Overall survival (OS) was calculated from the start date of radiotherapy to death or last follow-up date. For patients alive on the data cutoff date, survival was censored at the last study follow-up date.

### 2.2. Statistical Analysis

Patient characteristics are described using the rates, means, and medians of continuous and categorical variables. Survival analysis was done according to the Kaplan–Meier method. In order to assess the effect of various variables on overall survival, we performed a Cox proportional hazards regression analysis. For the multivariate analysis, we used known prognostic factors (age, clinical tumor and nodal stage, grading, and subtype) as well as significant factors from the univariate Cox regression analysis. Variables were entered simultaneously into the model. For the analysis of collinearity, we measured the variance inflation factor and kept the most clinically relevant variables.

Furthermore, we aimed to compare the effect of a pCR on OS in various subgroups including the breast cancer subtypes and chemotherapy regimens used. Two-sided *p*-values below the threshold of 0.05 were considered to be statistically significant. We performed all statistical analyses using SPSS (IBM Corp. Released 2016. IBM SPSS Statistics for Windows, Version 27.0. Armonk, NY, USA: IBM Corp.) Figures and were created using Microsoft Excel for Microsoft Office 365 Pro Plus (Redmond, Washington, WA, USA).

## 3. Results

An overview of the included trial population is shown in Table 1. The patient characteristics of the majority of the include population were previously described [13]. Median follow-up for the entire trial group was 20 years and 24.6 years for surviving patients. Breast-conserving surgery was performed in 178 (50.0%) patients, while 178 (50.0%) underwent mastectomy. The primary tumor location was on the right side in 156 (44.7%) patients and on the left side in 179 patients (49.4%). A total of 54.5% of the patients had grade 3 tumors and 66.5% had clinical T3–4 tumors. Half of the patients were clinically node positive (49.9%). The rates of the breast cancer subtypes were luminal A-like in 14.6% of patients, luminal B-like in 47.2%, HR+/HER2+ in 7.3%, HR−/HER2+ in 7.0%, triple negative in 17.1%, and of unknown subtype in 6.7%.

The mean interval between the start of radiotherapy and resection was 193 days, with a standard deviation of 80 days. Radiotherapy to the whole breast was given with either photons or cobalt radiation and regional nodal irradiation was performed in 84.8% with different target volumes. Tumor bed boosts were given in the majority of patients (95.5%), using brachytherapy + hyperthermia in 30.3%. The 2 Gy equivalent dose to the tumor bed ranged between 48.6 Gy and 75.5 Gy with a mean dose of 64 Gy.

Cytotoxic systemic therapy was administered in 95.8% of patients, of which 98 patients (27.5%) received a “standard” regime and 243 (68.3%) received a “substandard” regime. Adjuvant chemotherapy was given in 44 patients (12.4%). Endocrine therapy was prescribed in 275 women (77.2%) using tamoxifen, aromatase inhibitors, ovarian suppression, or surgical castration.

Table 2 shows the response parameters to the combined preoperative radiotherapy and systemic therapy. Out of the 356 patients, 15 women had an axillary lymph node dissection before the start of chemotherapy or radiation therapy. These women were excluded for the assessment of pCR and nodal response.

**Figure 1:**
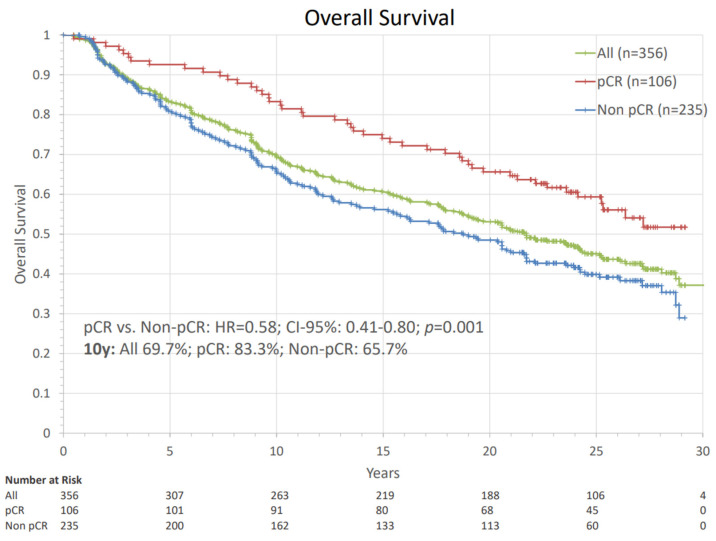
Overall survival of the whole cohort and groups separated by pathological complete response. The comparison using Cox regression analysis is presented with hazard ratio, the corresponding confidence interval, and *p*-value. Patients with ALND before systemic therapy were excluded from pCR analysis. pCR: pathologic complete response, HR: hazard ratio, ALND = axillary lymph node dissection, n: number of patients.

Figure 1 shows the survival curves according to the estimation by Kaplan–Meier statistic for all patients in the cohort and according to pCR status. 10-year, 15-year, 20-year and 25 year OS were 69.7%, 60.6%, 53.1% and 45.1% in the whole cohort. Patients that were diagnosed with a pCR had a significantly higher survival probability (HR = 0.58; CI-95%: 0.41–0.80; *p* = 0.001), which persisted over the observation period. After a 20-year follow-up, 65.6% diagnosed with a pCR were still alive, compared to 48.8% without pCR.

**Figure 2:**
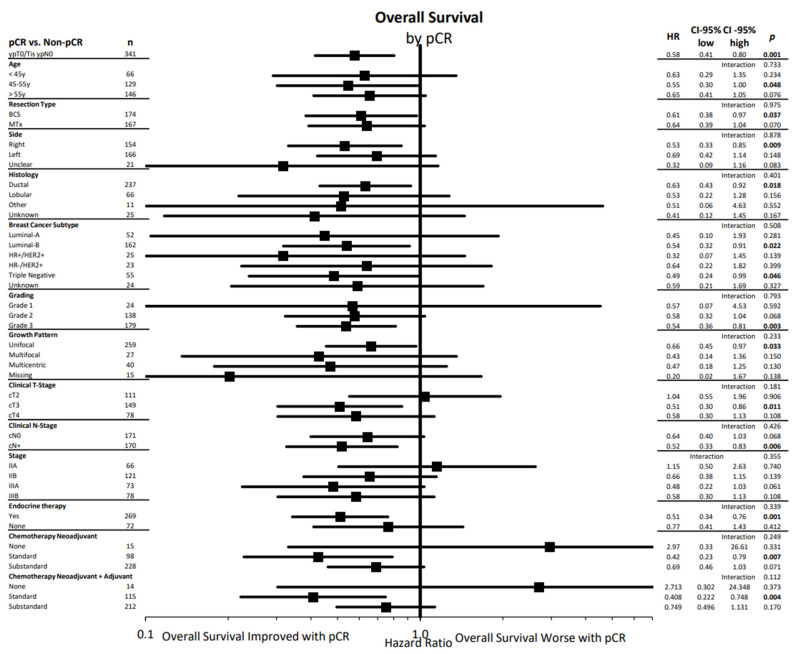
Cox regression analysis of overall survival by pCR-status (yes vs. no) status in different subgroups with hazard ratios, their corresponding confidence intervals and fitting interaction tests presented in a forest plot. pCR: pathologic complete response, BCS: breast-conserving surgery, MTx: mastectomy, HR+: hormone receptor positive, HER2: human epithelial growth factor receptor 2, c: clinical, T: tumor, N: nodal, G: grading, HR: hazard ratio, CI: confidence interval. Statistically significant differences are marked in bold.

Figure 2 summarizes the effect of pCR on overall survival in different subgroups. The positive effect of a pCR was evident in all investigated subgroups (age, resection type, primary tumor side, histology, breast cancer subtype, grading, growth pattern, clinical T- and N- Stage, tumor stage, and use of endocrine or chemotherapy therapy) with no significant interaction tests.

**Figure 3:**
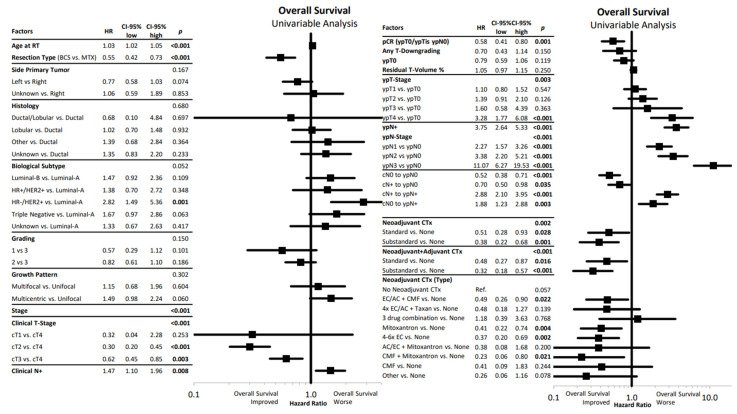
Univariate Cox regression analysis of overall survival with hazard ratios and their corresponding confidence intervals presented in a forest plot. RT: Radiotherapy, BCS: breast-conserving surgery, MTx: mastectomy, HR+: hormone receptor positive, HER2: human epithelial growth factor receptor 2, TN: triple negative, G: grading, HR: hazard ratio, CI: confidence interval, pCR: pathologic complete response, c: clinical, T: tumor, N: nodal, CTx: chemotherapy, AC: adriamycin/cyclophosphamide, EC: epirubicine/cyclophosphamide, CMF: cyclophosphamide, methotrexate, fluorouracil. Statistically significant differences are marked in bold.

Furthermore, we selected various factors that might influence survival in the investigated cohort. The univariate Cox regression analysis is shown as a forest plot in Figure 3. Higher age, mastectomy, higher clinical stage, clinical node positivity, pathological tumor and node positivity, failure of pathological complete response, and omission of chemotherapy were associated with worse OS.

**Figure 4:**
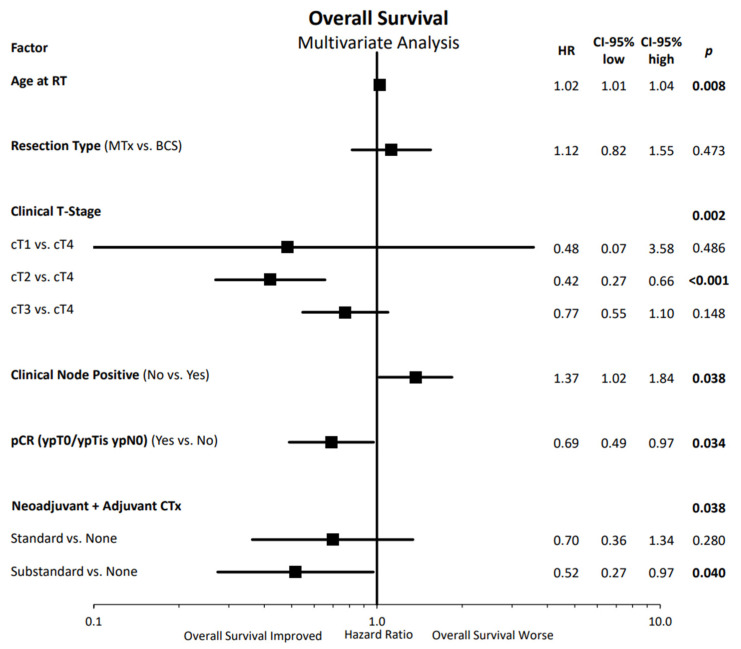
Multivariate Cox regression analysis of overall survival with hazard ratios and their corresponding confidence intervals presented in a forest plot. RT: radiotherapy, CTx: chemotherapy, BCS: breast-conserving surgery, MTx: mastectomy, pCR: pathologic complete response, T: tumor, G: grading, HR: hazard ratio, CI: confidence interval. Pathological complete response in the primary tumor and axillary lymph nodes was diagnosed in 106 patients (31.1%) with 15.4% in HR+ luminal A, 25.0% in HR+ luminal B, 30.8% in HR+/HER2+, 30.8% in HR−/HER2+ and 41.0% in triple-negative tumors. Statistically significant differences are marked in bold.

In the multivariate assessment of OS, shown in Figure 4, we detected improved OS with lower age, lower clinical tumor stage (significant for cT2 vs. cT4 stage), clinical node negativity, pathological complete response after neoadjuvant therapy, and with the use of chemotherapy (significant for substandard chemotherapy vs. no chemotherapy).

Nodal response assessment to neoadjuvant therapy documented that cN0 to ypN0 was present in 137 women (40.2%), cN0 to ypN+ in 34 women (10.0%), cN+ to ypN0 in 99 women (29.0%), and cN+ to ypN+ in 71 women (20.8%). The mean reduction in tumor diameter measured by ultrasound compared to the pathological tumor diameter after resection was 45.4 mm. When idealizing the tumor as a spherical volume before and after naRCT the mean and median residual volumes were 13% and 0.2%.

**Figure 5:**
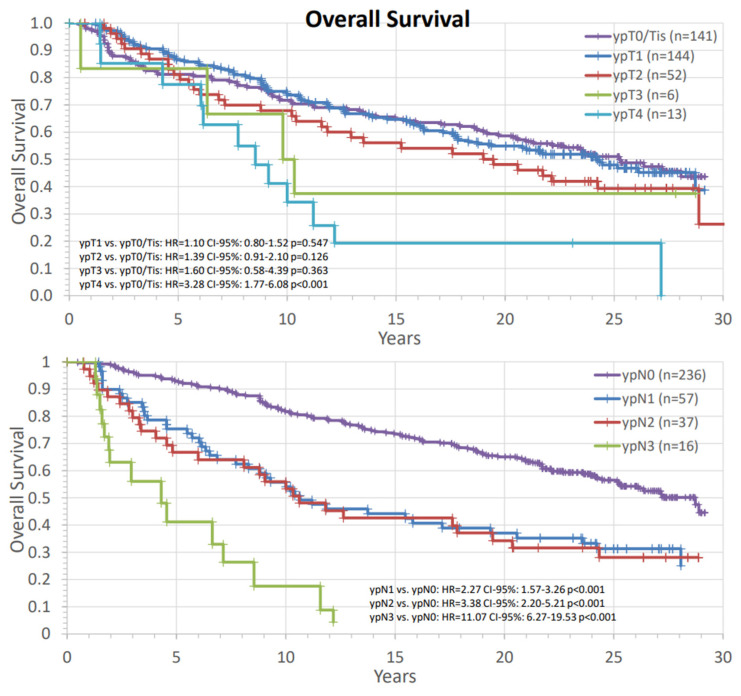
Analysis of survival of different postoperative T-stages and N-stages. The comparisons are presented with hazard ratios and their corresponding confidence intervals and *p*-values against ypT0/Tis and ypN0. HR: hazard ratio, ypT: postoperative primary tumor stage, ypN: postoperative nodal stage, CI: confidence interval, n: number of patients.

Figure 5 assessed the survival rates in different response groups to the preoperative therapy according to primary tumor and nodal status. The risk of death was numerically higher in all groups with residual tumor in the breast. The comparisons were significant for ypT4 vs. ypT0/Tis. The amount of residual cancer in the lymph nodes after naCT and naRT was also associated with worse survival in our analysis.

**Figure 6:**
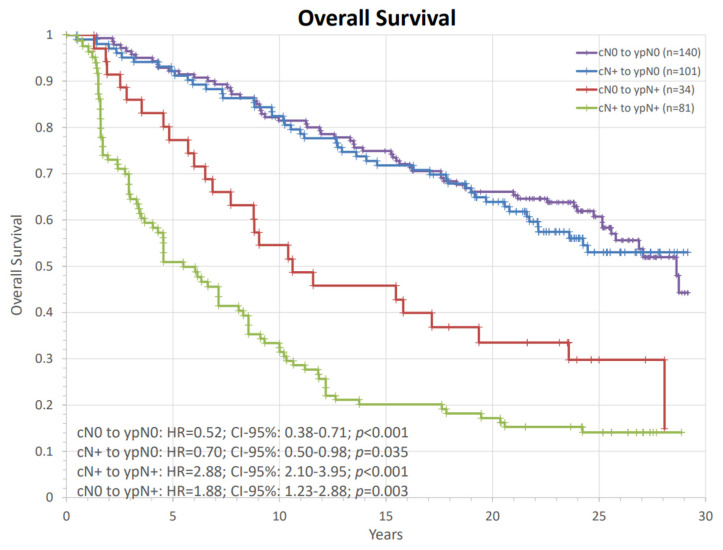
Analysis of the impact of clinical and pathological nodal status on overall survival. The comparisons of each group are set against the remaining patients in the whole cohort and are presented with hazard ratios and their corresponding confidence intervals and *p*-values. HR: hazard ratio, ypT: postoperative primary tumor stage, ypN: postoperative nodal stage, CI: confidence interval, pCR: pathological complete response, n: number of patients.

Figure 6 showed the long-term survival according to nodal response, demonstrating that both groups with ypN0 status had favorable survival compared to node positivity after primary systemic therapy and radiotherapy. Patients with pathological node negative status had similar long-term survival, regardless of primary clinical status.

**Figure 7:**
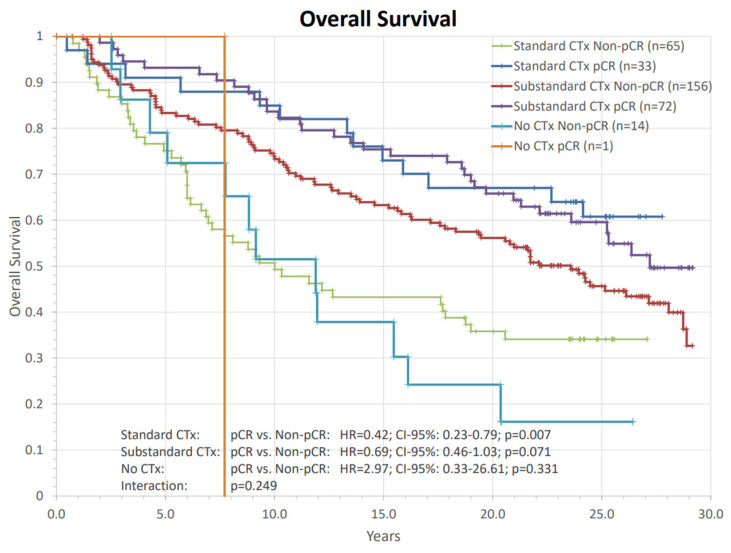
Analysis of the impact of pathological complete response (pCR) on overall survival between different preoperative chemotherapy regimens. The comparisons are presented with hazard ratios and their corresponding confidence intervals and *p*-values. HR: hazard ratio, CTx: chemotherapy, CI: confidence interval, pCR: pathological complete response, n: number of patients.

In both categories of neoadjuvant systemic therapy, “standard” protocol or “substandard” protocol pCR was numerically favorably associated with OS (Figure 7). This comparison was statistically significant for patients receiving a standard chemotherapy schedule (Standard CTx: HR = 0.42; CI-95%: 0.23–0.79; *p* = 0.007; substandard CTx: HR = 0.69; CI-95%: 0.46–1.03; *p* = 0.071; No CTx: HR = 2.97; CI-95%:0.33–26.61; *p* = 0.331). Again, we did not find a significant interaction between the chemotherapy regime and pCR on OS (*p* = 0.249).

**Figure 8:**
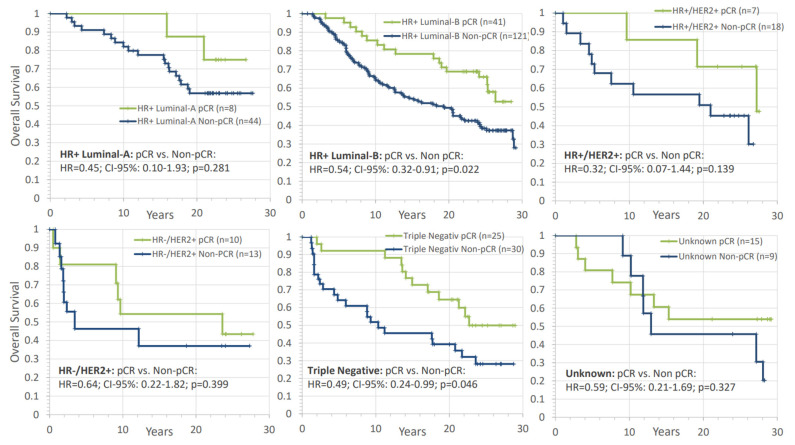
Analysis of the impact of pathological complete response (pCR) on overall survival in different breast cancer subtypes. The comparisons are presented with hazard ratios and their corresponding confidence intervals and *p*-values. HR+: hormone receptor positive, HER2+: human epithelial growth factor receptor 2, pCR: pathologic complete response, HR: hazard ratio, n: number of patients.

Next, we further assessed whether pCR achieved by combined naCT and naRT is prognostic in different subgroups. According to Figure 8, pCR was a positive prognostic factor in all investigated biologic subtypes, although statistical significance was only reached for the luminal-B like and triple-negative tumor subgroups (HR+ luminal B-like: HR = 0.54; CI-95%: 0.32–0.91; *p* = 0.022; triple negative: HR = 0.49; CI-95%: 0.24–0.99; *p* = 0.046). We did not find a significant interaction between pCR and biological subtype (*p* = 0.508).

## 4. Discussion

The results of this cohort including patients with high-risk breast cancer treated with naCT and naRT followed by breast-conserving surgery or mastectomy showed acceptable survival rates, with 53.1% being alive after a 20-year follow-up. Long-term adverse events were also reported to be rare and cosmetic outcomes and quality of life assessments were favorable [25,26].

### 4.1. PCR as a Prognostic Factor

Our results demonstrate that pCR following naRCT was an independent prognostic factor for long-term overall survival across all investigated subgroups including different histological entities, biological subtypes, stages, growth patterns, and systemic agents.

Two large meta-analyses have already described the value of pCR for long-term mortality according to different subtypes in women treated with neoadjuvant systemic therapy [3,4]. Minckwitz and colleagues reported a trend to higher impact of pCR on OS with older age, more advanced nodal burden, and more biologically aggressive subtypes such as those with ductal histology and higher grades as well as those that are hormone receptor negative and/or HER2 enriched. Similarly, the CTNeoBC meta-analysis demonstrated that pCR had no significant effect in the subgroup analysis in T1-tumors, lobular histology, grade I, and hormone receptor-positive disease, demonstrating a lower impact in less aggressive diseases. Our subgroup results are similar regarding tumor and nodal status as well as lower grading. Expectantly, women with more aggressive tumors were more likely to achieve a pCR; however, we found no impact of lobular histology and biological subtype when these women had a pCR, compared to other groups. Women with HR+ luminal B-like and triple-negative tumors had a statistically significant benefit from pCR, but numerically, all subgroups benefited from a pCR, even the luminal A-like tumors. The reasons for these discrepancies could be due to the addition of radiotherapy, limited sample size, or suboptimal systemic therapy. The pCR rates in the ctNeoBC analysis are reported to be 7.5% for HR+/HER2- grade 1/2, 16.2% for HR+/HER2- grade 3, 18.3% for HR+/HER2+ without trastuzumab, 30.2% for HR−/HER2+ without trastuzumab, and 33.6% for the triple-negative subgroup. The respective rates in our cohort are 15.4%, 25.0%, 30.8%, 40.0%, and 41.0%. These numbers compare favorable for the addition of radiotherapy in all cohorts.

### 4.2. Chemotherapy Regimens

The administered chemotherapy regimens in our cohort were inferior to the current standard of care with only 98 women (27.5%) receiving a currently acceptable cytotoxic therapy protocol. With the use of modern systemic regimes including intensive cytotoxic agents, HER2+ targeted agents, as well immune checkpoint inhibitors, the current pCR rates are as high as 46–68% for HER2+ disease [27,28,29] and as high as 64.8% for triple-negative disease [30].

The overall survival rates in our cohort after 10 y with 69.5% and 15 years with 60.5% are comparable to the meta-analysis which assess pre- vs. postoperative survival rates (10 y: 69% and 15 y: 59.1%), despite a clinically more unfavorable prognosis regarding grading, primary tumor size, and nodal status in our cohort [2].

The multivariate assessment demonstrated that age, clinical T- and N-stage, chemotherapy, and pCR status had a significant impact on mortality. Residual lymph nodes were the numerically strongest negative predictor for OS. This is in accordance with the data reported in naRT [31] and naCT alone [4]. Pathological nodal positivity had a worse prognosis, irrespective of primary tumor response. It has also been recently recognized that residual cancer burden (RCB) in primary tumor and lymph nodes can further differentiate patients into different prognostic groups [32]. Similarly, we also detected a relationship between the residual tumor in the breast and lymph nodes and mortality based on pathological T and N status as shown in Figure 4.

### 4.3. The Role of Radiotherapy

A large comparable cohort with long-term follow-up was reported from “Gustave Roussy” hospital in France, where 187 patients were treated with naRT without additional systemic therapy. This is in contrast to our cohort, where only 3.5% received no naCT. Tumor resection was performed after an interval of four weeks, while our cohort underwent resection after a median of 28.9 weeks. These variables may explain the differences in the pCR rate of 10% vs. 29.2%. Similar to our report, the pCR rate was highest in TN tumors (26% and 40%). The long-term survival results of our patients compare favorably to this publication, with 10-year and 20-year OS rates of 55% and 41%, reflecting the advances over time in high-risk breast cancer. Other research groups found similar rates of pCR, where the range after preoperative radio- or radiochemotherapy was 16 to 45% [13,33,34,35,36,37,38,39].

The most recently published study in this field is the small prospective PRADA trial. This study investigated preoperative chemotherapy followed by radiotherapy with an interval of 2–6 weeks, followed by skin-sparing mastectomy and DIEP flap reconstruction [40]. This approach was found to be feasible with wound complications similar to postoperative approaches. Breast and nodal pCR rates were 21% and 26%, which also varied by subtype. These modest rates might be a result of the inclusion of advanced stages and a predominance of HR+ tumors.

Another recent publication reported on 153 prospectively observed patients with locally advanced breast cancer treated with naRT and naCT [41]. Overall, 68% were stage III, with all patients undergoing mastectomy. Miller–Payne score 5 was achieved in 19% of HR+, 82% of HER2+, and 55% of TN patients, suggesting that current systemic agents substantially increase the response rates in HER2+ and moderately in TN disease.

With a shortage of evidence from randomized studies, the gain in pCR rate driven by naRT can only be indirectly deduced from published works. A retrospective single-center comparison reported a numerically higher pCR rate in the patients treated with naRT and naCT compared to the standard sequence (naCT followed by surgery and RT) of 39% vs. 18% in the standard sequence, with especially high rates of pCR in the triple-negative cohort (78% vs. 27.6%) [42].

Modern prospective studies have reported a high percentage of patients with a pCR, especially in HER2+ disease. However, retrospective data suggest that naRT might even add to these high pCR rates, though the absolute gain is less than in other subtypes [43]. Triple-negative disease also reports pCR rates around 50%, yet naRT could substantially advance these results with improvements of 25% reported for naRT alone [31]. Another interesting option is naRT in luminal subtype, where the pCR rates are traditionally modest. Here, the combination of naRCT has been reported to achieve pCR in 48% of patients [43]. It would also be intriguing to analyze biological mechanisms in response to naCT and naRT to identify patients who would benefit from naRT. These questions, however, can only be answered in an adequately powered, randomized trial comparing adjuvant radiotherapy to naRT in the setting of naST [37].

The value of the addition of cytotoxic therapy in the preoperative treatment has been demonstrated in a small randomized trial where naCT added to naRT was associated with improved DFS and OS in stage IIb–IIIa breast cancer compared to naRT alone.

Long-term overall survival in neoadjuvant vs. adjuvant radiotherapy appears to be similar according to one randomized trial [9,10] and a large registry trial [44]. Beyond inoperable cases or an improved ability to perform breast conservation surgery, response to naCT offers the possibility to tailor post-neoadjuvant therapy according to the treatment response [5,45,46]. The addition of radiotherapy to the preoperative therapy paradigm offers the advantages of treating the tumor in situ with improved target localization, the possibility of improved side effects due to smaller target volumes, and surgical removal of the tissue treated with the highest dose, improving reconstruction results and allowing for early translational and clinical analysis of response. As systemic agents affecting locally and distantly located tumor cells usually drive pCR, the value of pCR achieved by the addition of a local therapy has been questioned. Here, we aimed to investigate the prognostic effect of pCR in this large cohort of high-risk women treated with combined chemotherapy and radiotherapy in the preoperative setting. 

Just as in breast cancer, complete responses achieved by naRT or naRCT have also been demonstrated to be highly prognostic in head and neck cancer [47], soft tissue sarcoma [48], and rectal cancer [49] regardless of the addition of cytotoxic agents. We believe that there is no reason to suggest that pCR driven by naRT or naCT has a differential prognostic impact on OS.

The importance of nodal response reinforces the standing of a thorough pre- and postoperative assessment of the axillary lymph nodes for the correct application of local therapy in this setting, including regional nodal irradiation, which is associated with a proven benefit in overall survival [50]. Because of the outdated radiation techniques used in the treatment of our cohort, the specific nodal levels cannot be reliably described. Nonetheless, due to known anatomical borders traditionally used for the field setup, we expect that large parts of the regional lymph node levels I–IV were treated with therapeutic doses.

### 4.4. Main Limitations

The main limitations of our analysis are in its inherent retrospective design as well as the outdated chemotherapy agents and radiotherapy techniques. No HER2-targeted therapy was used, limiting the generalizability of the findings in this subgroup to the current standard of care. In addition, the histological assessment of Ki-67 was not standardized and thus no categorization into the currently used subgroups was possible. The current analysis of nodal response includes frozen section assessment for the detection of micrometastases and isolated tumor cells which was not performed at our institution during the trial period. This might have resulted in a lower detection rate of low-volume nodal disease.

Another aspect to consider when analyzing the pCR rate with the addition of naRT is the interval between radiotherapy and surgery. A longer waiting time after RT is usually associated with a higher pCR rate. However, even the relatively short interval of 6 weeks showed some complete responses in patients treated with naRT alone [31]. Furthermore, inter-observer variability of the diagnosis of a pCR has also been described in the literature [51].

## 5. Conclusions

Neoadjuvant systemic therapy and radiotherapy prior to surgical resection achieved good long-term survival in high-risk breast cancer. Radiotherapy-influenced pathological complete response maintains its prognostic value in various breast cancer subtypes and different subgroups.

## Figures and Tables

**Table 1:** Overview of the baseline and treatment characteristics of the patient cohort. Data are presented as absolute numbers and percentages.

Characteristics	N (%)	Characteristics	N (%)	Characteristics	N (%)
Median FU [y]	20.4	**Clinical Tumor Stage**	356	**Resection type**	356
Median Age [y]	53.5	cT1	3 (0.8%)	BCS	178 (50.0%)
Age < 45 y	70 (19.7%)	cT2	116 (32.6%)	Mastectomy	178 (50.0%)
Age 45 y–55 y	135 (37.9%)	cT3	155 (43.8%)	**Neoadjuvant Systemic Therapy**	356
Age > 55 y	151 (42.4%)	cT4	82 (23.0%)	No Neoadjuvant Systemic Therapy	15 (4.2%)
**Side Primary Tumor**	356	**Clinical Nodal Stage**	341	Agent Concomitant to RT	122 (34.3%)
Right	159 (44.7%)	cN0	171 (50.1%)	**“Standard” Regime**	98 (27.5%)
Left	176 (49.4%)	cN+	170 (49.9%)	AC/EC + Taxane	11 (3.1%)
Unknown	21 (5.9%)	**Clinical Stage**	356	AC/EC + CMF	82 (23.0%)
**Histological type**	356	I	1 (0.3%)	Combination of “Standard” Regime + One Agent	5 (1.4%)
Ductal	247 (69.4%)	IIA	68 (19.1%)	**“Substandard” Regime**	243 (68.3%)
Lobular	66 (18.5%)	IIB	124 (34.8%)	Mitoxantrone	109 (30.6%)
Mixed ductal/lobular	2 (0.6%)	IIIA	80 (22.5%)	AC/EC (4–6 cylces)	113 (31.7%)
Other	13 (3.7%)	IIIB	82 (23.0%)	AC/EC + Mitoxantrone	4 (1.1%)
Unknown	28 (7.9%)	IIIC	1 (0.3%)	CMF (3–6 cycles)	4 (1.1%)
**Histological Grading**	356	**Growth pattern**	356	CMF + Mitoxantrone	7 (2.0%)
Grade 1	24 (6.7%)	Unifocal	272 (76.4%)	Other (Taxane; Epirubicine + Taxane; EC + Vinorelbine; Epirubicine + Taxane + CMF)	6 (1.7%)
Grade 2	138 (38.8%)	Multifocal	27 (7.6%)	**Additional Adjuvant Systemic Therapy**	44 (12.4%)
Grade 3	194 (54.5%)	Multicentric	41 (11.5%)	2–4x EC	3 (0.8%)
**Breast Cancer Subtype**	356	Unknown	16 (4.5%)	3–6x CMF	27 (7.6%)
HR+ Luminal A-like	52 (14.6%)	**Radiation Treatment Details**	356	2–4x Taxane	10 (2.8%)
HR+ Luminal B-like	168 (47.2%)	Mean Time Interval RT to Rx [days]	193; SD = 80	Other (CMF + Taxane; Epirubicine + Taxane; EC + Taxane)	5 (1.4%)
HR+/HER2+	26 (7.3%)	Mean Tumor Bed Dose as EQD2 (3.7)	64 Gy; Range: 48.6–75.5 Gy	**Neoadjuvant + Adjuvant Systemic Therapy**	
HR−/HER2+	25 (7.0%)	Regional Nodal Irradiation	302 (84.8%)	“Standard” Regime	113 (32.0%)
Triple Negative	61 (17.1%)	Brachytherapy Boost + Hyperthermia	108 (30.3%)	“Substandard” Regime	229 (64.0%)
Unknown	24 (6.7%)	Tumor Bed Boost	340 (95.5%)	**Endocrine Therapy**	275 (77.2%)

**Table 2:** Overview of the response parameters of the patient cohort. Data presented as absolute numbers and percentages.

Response Parameters	N (%)	Response Parameters	N (%)
Neoadjuvant Therapy followed by breast operation and ALND	341 (95.8%)	**Breast Cancer Subtype**	**pCR**
pCR (ypT0/Tis and ypN0)	106 (31.1%)	• HR+ Luminal A-like	8 (15.4%)
**Pathological Tumor Stage**	356	• HR+ Luminal B-like	42 (25.0%)
ypT0	125 (35.1%)	• HR+/HER2+	8 (30.8%)
ypTis	16 (4.5%)	• HR−/HER2+	10 (40.0%)
ypT0/Tis	141 (39.6%)	• Triple Negative	25 (41.0%)
ypT1	144 (40.4%)	• Unknown	15 (62.5%)
ypT2	52 (14.6%)	**Response to Neoadjuvant Therapy**	
ypT3	6 (1.7%)	Any Primary Tumor Downstaging	328 (92.1%)
ypT4	13 (3.7%)	cN0 to ypN0	137 (40.2%)
**Pathological Nodal Stage**	341	cN+ to ypN0	99 (29.0%)
ypN0	236 (69.2%)	cN0 to ypN+	34 (10.0%)
ypN1	57 (16.7%)	cN+ to ypN+	71 (20.8%)
ypN2	32 (9.4%)	Mean T-Size Reduction [mm]	45.4
ypN3	16 (4.7%)	Mean Residual Tumor Volume [%]	13.0
**Axillary Lymph Node Dissection**		Median Residual Tumor Volume [%]	0.2
Mean Number of dissected Nodes	19		
Mean Number of + Nodes	7		

## Data Availability

The data presented in this study are available in this article.

## Abbreviations

AC	adriamycin, cyclophosphamide
ALND	axillary lymph node dissection
BCS	breast-conserving surgery
CTx	chemotherapy
CI	confidence interval
CMF	cyclophosphamide, methotrexate, fluorouracil
DFS	disease free survival
DIEP	deep inferior epigastric perforator
EC	epirubicin, cyclophosphamide
ER	endocrine receptor
EQD2	2 Gy equivalent dose
FU	follow-up
HER2	human epidermal growth factor receptor 2
HR	hazard ratio
ICI	immune checkpoint inhibitor
IMN	internal mammary nodes
MTx	mastectomy
naCT	neoadjuvant chemotherapy
naRCT	neoadjuvant radiochemotherapy
naRT	neoadjuvant radiotherapy
naST	neoadjuvant systemic therapy
OS	overall survival
pCR	pathologic complete response
RCB	residual cancer burden
RNI	regional nodal irradiation
RT	radiotherapy
SLND	sentinel lymph node dissection
ST	systemic therapy
TN	triple negative
ypN	postoperative nodal stage
ypT	postoperative primary tumor stage
